# Assessing How Participators Combine Acts in Their “Political Tool Kits”: A Person-Centered Measurement Approach for Analyzing Citizen Participation

**DOI:** 10.1007/s11205-016-1364-8

**Published:** 2016-05-30

**Authors:** Jennifer Oser

**Affiliations:** Department of Politics and Government, Ben-Gurion University of the Negev, Building 72, 6th floor, 8410501 Beer Sheva, Israel

**Keywords:** Citizen participation, Political participation, Citizenship norms, Latent class analysis, Participatory inequality

## Abstract

Scholars have recognized that a recent increase in the ways citizens participate beyond the electoral arena may be a promising avenue of renewal for citizen participation. In this article we test the theory that different kinds of citizenship norms motivate some citizens to specialize in electoral-oriented activities (e.g. voting), while others specialize in non-institutionalized activities (e.g. protest). The latent class analysis of data from the U.S. Citizen, Involvement and Democracy Survey (2005) in the current study assesses how actors combine a variety of acts in their “political tool kits” of participation, and facilitates a comparison to prior findings that analyze single political behaviors. Results indicate a participatory type that specializes in non-institutionalized acts, but the group’s high probability of voting does not align with the expectations in the literature. An electoral-oriented specialist type is not identified; instead, the findings show that a majority of the population is best characterized as disengaged, while a small group of all-around activists embrace all possible opportunities for political action. The actor-centered theoretical and measurement approach in this study identifies caveats to the theory that changing citizenship norms are leading to civic and political renewal. We discuss the implications of these findings for measuring different aspects of democratic (dis)engagement and participatory (in)equality.

## Introduction

Although citizen participation is often viewed as an instrument for achieving civic and political aims, it is also considered an intrinsic democratic good that impacts on various aspects of citizens’ quality of life (Arendt 1958; Barber 2003; Pateman 1970, 2012). Empirical research has shown that the health and quality of democracy are affected by patterns of political participation (Morlino 2011), and a perennial topic of interest in this field of study has been various aspects of participatory inequality (Dahl 2006; Lijphart 1997; Schlozman et al. 2012; Verba et al. 1995).

A vibrant debate has taken place in recent years, however, regarding the implications of citizen participation trends in contemporary democracies. On the one hand, there is consensus that electoral-oriented political participation has generally stagnated or declined in advanced democracies in the past few decades. What has been described as a “transformational” school of thought (Copeland 2014a) highlights a different trend: namely, that during the same time period additional, non-electoral-oriented political acts have become more common (e.g., Bennett 2012; Bolzendahl and Coffé 2013; Dalton 2008, 2015; Inglehart 1997; Norris 2002; Welzel 2013; Zukin et al. 2006). These studies share a central argument that broadening the empirical lens to include the rich diversity of contemporary political behavior beyond electoral-oriented actions reveals a renewal of political engagement, particularly among contemporary youth.

A prominent theory in this line of research is that the increased prevalence of new kinds of pro-democratic citizenship norms, particularly among young and socio-economically advantaged citizens, is responsible for the recent stagnation and decline of electoral-oriented participation and the simultaneous rise in citizen participation beyond the electoral arena. The act of voting plays a central role in the literature on this topic. Although some studies have highlighted voting data that suggest a pattern of stagnant fluctuation rather than decline, the main theoretical argument of the transformational school of thought does not hinge on challenging the empirical analysis of voting trend data. Rather, the transformational argument is a novel theory about how changing citizenship norms motivate individuals to choose to engage in some types of political activity (e.g. non-institutionalized acts) but not others (e.g. electoral-oriented acts).

In the current study we test the theory that citizenship norms may motivate some individuals to specialize in certain types of citizen participation. As the variety and prevalence of political acts beyond the electoral arena have increased—including for example, internet activism, protest activities and political consumerism—a common theme in scholarship on the rise and fall of different kinds of political activities is that contemporary young people are politically active, but that they focus their energies beyond the electoral arena. Prior research has not yet tested how individual citizens combine sets of activities in their individual-level political repertoires. A measurement approach that analyzes *actor*-*centered repertoires* of political action—in contrast to a common approach of analyzing *separate political acts*—is required to test the theory of civic and political renewal that is increasingly prominent in the literature.

To assess the transformational school’s theory, it is useful to re-examine established findings that focus on separate political behaviors by analyzing the same data with a measurement approach that identifies actor-centered repertoires. In this article we therefore re-examine a prominent study on this topic (Dalton 2008) to test hypotheses about the relationship between citizenship norms and distinct types of political participators. To identify distinct types of participators, we use latent class analysis, a technique that is particularly well suited for identifying how individuals combine a range of political acts in their individual-level “toolbox” of political behavior. The findings show that a participator type is identified that specializes in non-institutionalized activity, but the group’s high probability of voting does not align with expectations in the literature. The results show no evidence of an electoral-oriented type, however; instead, the findings indicate that a majority of citizens are best characterized as disengaged, while a small group of all-around activists embrace all possible opportunities for political action.

The actor-centered theoretical and measurement approach used in the current study therefore identifies caveats to prior findings regarding the types of political participators that engage in civic and political activity, as well as their normative and socio-demographic correlates. These findings are instructive from a measurement perspective for researchers who aim to assess how individuals are combining (or not combining) newly emerging types of citizen participation, such as Facebook activism and buycotting, with more traditional political activities such as voting. From a substantive perspective the findings support observations in the literature that changing citizenship norms and citizenship participation trends may increase participatory inequality in various ways. The concluding discussion expands upon these measurement and substantive implications.

## Literature

### Trends in Citizen Participation

There is general consensus in the literature that turnout in advanced democracies has, at best, stagnated since the 1950s despite institutional and socio-economic changes that would be expected to increase voting levels (Blais 2010; Blais et al. 2004; Dalton 2008; Franklin 2004; Lewis-Beck et al. 2008; McDonald 2010, 2015; Norris 2002; Wattenberg 2012). This consensus, coupled with evidence of weakening political parties and lower levels of political campaign-related activity (Dalton and Wattenberg 2000; Van Biezen et al. 2012; Van Biezen and Poguntke 2014; Whiteley 2011) indicate that traditional electoral-oriented actions have not been a source of political rejuvenation in recent years.

A consensus has also emerged, however, that some forms of non-institutionalized participation are on the rise during this same time period (Christensen 2014; Copeland 2014a; Dalton 2008; Inglehart and Catterberg 2002; Norris 2002; Stolle et al. 2005; Van Deth et al. 2007). The civic and political participation of young people has gained particular attention in this regard (Amnå 2012; Amnå et al. 2009), with some authors highlighting the decline of youth participation in electoral and institutional-oriented participation (Blais et al. 2004; Fieldhouse et al. 2007; Phelps 2012; Wattenberg 2012), and others emphasizing the increase in young citizens’ activism beyond the electoral arena (Dalton 2015; Martin 2012; Norris 2002; Quaranta 2016; Sloam 2012, 2013, 2014; Vromen et al. 2015). Drawing on single-country data for the United States from some of the most credible academic surveys of political behavior since the 1950s, Dalton (2008, p. 90) shows a clear increase in political participation beyond the electoral arena—including acts that were previously described as “unconventional” (Barnes et al. 1979) such as petitioning and protesting, as well as newly emerging political activities such as internet activism and political consumerism.

One prominent explanation of these shifting patterns of participation is that changing citizenship norms, particularly among young people, are transforming global patterns of political behavior (Bennett 2012; Bennett, Wells and Freelon 2011; Copeland 2014b; Dalton and Welzel 2014; Jennings 2015; Theocharis 2011a, b; Van Deth 2007, 2012; Welzel 2007, 2013). Increases in the prevalence of these citizenship norms—variously referred to as “engaged” (Coffé and van der Lippe 2010; Dalton 2008, 2015), “critical” (Geissel 2008; Norris 1999, 2011), “self-actualizing” (Bennett 2008, 2012; Shehata et al. 2015; Wells 2014), and “emancipative” (Welzel 2007, 2013)—are identified as motivating increasingly common acts of non-institutionalized participation. Cross-national empirical investigations of these claims indeed suggest that a decline in duty-based norms has led to decreased institutional participation such as voter turnout (Blais and Rubenson 2013), while certain types of “good citizenship” norms are associated with some non-institutionalized activities such as protest (Welzel and Deutsch 2012), albeit with weaker effects in new and less developed democracies (Bolzendahl and Coffé 2013).

From a theoretical perspective, the studies identified by Copeland (2014a) as belonging to the transformational school of thought were not the first studies to highlight the need to look beyond the electoral realm in order to understand changing patterns of citizen participation. Indeed, over 40 years ago, Verba et al. emphasized the need to “expand the view of participation to a wider range of activity” (1971, p. 19), and Barnes et al.’s Political Action Study (1979) expanded the focus even further by investigating protest activities that had become more prevalent in the 1960s and 1970s. A new theoretical contribution of the transformational school of thought, however, is the argument that changing citizenship norms motivate individuals to specialize in certain types of participation (e.g. non-institutionalized activities) while simultaneously turning away from others (e.g. electoral-oriented activities) (Bennett et al. 2011; Copeland 2014b; Dalton 2008, 2015; Dalton and Shin 2014; Dalton and Welzel 2014; Welzel 2013).

The motivating theory in this line of the literature about the relationship between citizenship norms and participation does not focus on the relationship between citizenship norms and a single political act (such as voting or protest) but rather proposes that actors combine a range of possible political acts in specific ways. In this literature, Dalton’s (2008) influential study on citizenship norms and expanded patterns of political participation in the United States is among the clearest in identifying the mechanisms behind the proposed political renewal of contemporary democracies. In the current study we therefore re-examine data used by Dalton (2008) in order to identify how an actor-centered theoretical and measurement approach adds a new perspective to extant findings. The following section therefore specifies the contrasting “ideal types” of political participators that, if found, would provide robust evidence that values change is leading to civic and political renewal.

### Norms and Behaviors of “Ideal Types” of Participators

Research has shown that, in a variety of democracies, a decline in norms of civic duty is related to a decline in voting (Blais et al. 2004; Blais and Rubenson 2013; Bowler and Donovan 2013; Raney and Berdahl 2009). Scholars who interpret recent participation trends as indicative of civic and political renewal have not disputed these findings, and Dalton’s (2008, pp. 89–92) interpretation of data on turnout in the U.S. is that voting has indeed declined somewhat since the 1950s. Dalton represents the transformational school of thought, however, in arguing that the decline in voting rates due to the decline in duty-based norms tells “only half of the story” (2008, p. 83) of how generational norm change has influenced patterns of political participation. The other half of the story according to the transformational school of thought is that the rise of engaged citizenship norms is yielding a new style of citizen engagement in which individuals are particularly active beyond the electoral arena.

Although scholars of these increasingly prevalent engaged citizenship norms and behavior have generally praised their emergence as a sign of democratic renewal (Bennett 2008, 2012; Norris 1999, 2011; Schudson 1998, 2000), Dalton has emphasized that it would be a mistake to think that “one set of norms is good, and the other is bad” (2008, p. 84). Instead, this line of the literature proposes that engaged and duty-based norms have contrasting emphases about the role of the democratic citizen, and the resulting political activities of those adhering to one norm versus the other. Dalton outlined the implications of each of these contrasting norms (engaged and duty-based) for the types of political participators that should be present in the population. To specify the implications of this argument, we propose that it is useful to conceptualize the participatory behavior motivated by engaged and duty-based norms as “ideal types” in the classic Weberian sense, as analytical constructs “formed by the one-sided *accentuation* of one or more points of view” (Weber 1949 [1904], p. 90).

In their ideal typical form, duty-based norms are expected to motivate a specific pattern of individual political action, described as follows: “Duty-based norms of citizenship encourage individuals to participate as a civic duty, which may stimulate election turnout and participation in other institutionalized forms of action” (Dalton 2008, p. 86). In other words, citizens with duty-based norms are not expected to be politically disengaged citizens who may vote but do little else; rather, citizens with duty-based norms are expected to attribute positive value to electoral-oriented activity in general. In addition to the expectation that citizens motivated by duty-based norms would have relatively high voting rates, they are also expected to take part in electoral-oriented participation, such as actively supporting electoral campaigns through activities like displaying campaign signs.

In contrast, engaged citizenship norms are expected to motivate opposite behavioral emphases: “Engaged citizenship should also stimulate political action. However, the expressive, participatory emphasis of these norms suggest a shift in the modes of participation—away from elections and party activity, seen as institutionalized expressions of citizen duty, and toward individualized and direct forms of action” (Dalton 2008, p. 86). In other words, although Dalton did not propose that engaged citizens would completely shun the act of voting or other electoral-oriented political acts, he expected that citizens motivated by engaged norms would pursue a combination of political acts that reflected a shift away from voting and elections, and toward non-institutionalized political action.

Although the ideal types of engaged and duty-based political participators described above are derived from Dalton’s (2008, 2015) scholarship and are widely referenced in the literature (e.g., Coffé and van der Lippe 2010; Copeland 2014b; Quaranta 2016), these typological concepts have not yet been tested with an actor-oriented measurement model. Rather, studies on the topic tend to analyze political acts either as single behaviors, or as separate additive indices of electoral versus non-electoral behavior (e.g., Bolzendahl and Coffé 2013; Marien et al. 2010; Martin 2012). It is noteworthy, however, that the transformational school’s theory of generational change relies on the existence of these kinds of specialist participator types. The predicted generational change of norms and behavior in Dalton’s research is based on the argument that the duty-based norms more common among an older generation of electoral-oriented participants will be replaced over time by the engaged norms of contemporary younger citizens who uniquely emphasize non-institutionalized participation. The implications for generational change on patterns of political behavior are summarized by Dalton as follows:

In short, the sky is not falling. Rather than an absolute decline in political action, the changing norms of citizenship are shifting the way Americans participate in politics—decreasing electoral participation, but increasing other forms of action (Dalton 2008, p. 91).

In sum, the transformational school of thought argues that two contrasting citizenship norms are each expected to motivate ideal types of political participators who combine political acts in distinctive ways: a “duty-based” type of participator who is relatively active in electoral-oriented and institutional political behavior (but relatively inactive in political action beyond the electoral arena); and conversely, an “engaged” type of participator who is relatively active in civic and political participation beyond the electoral arena (but relatively inactive in electoral-oriented participation).

## Research Design and Hypotheses

### A Political “Toolbox” Research Design

To clarify the unique contribution of the transformational school of thought in the literature, it is useful to review two broad categories of political acts often discussed in comprehensive reviews of participation scholarship: first, participation described as “electoral-oriented”, “institutionalized”, or “conventional”; and second, participation characterized as “non-electoral”, “non-institutionalized”, or “unconventional” (e.g., Brady 1999, p. 767; Harris and Gillion 2010, pp. 145–150; Marien et al. 2010, pp. 187–188; Marsh and Akram 2015; Quaranta 2016; Vráblíková 2014).^1^ Additional categories have certainly been identified in the literature, including Verba et al. (1978) identification of four main modes of institutional-oriented participation, Teorell et al. (2007) analysis of a more diverse range of political acts that identified five main modes of participation, and Talò and Mannarini’s (2015) implementation of Ekman and Amnå’s (2012) four-part typology of political participation and civic engagement.^2^ As the variety and creativity of political acts has increased, especially into the realm of online participation, conceptual approaches have been developed to categorize different types of political acts (Gibson and Cantijoch 2013; Theocharis 2015; Van Deth 2011, 2014). Despite the development of more fine-grained conceptual approaches, theoretical arguments regarding the two main broad categories of electoral-oriented versus non-institutionalized political behavior persist in the research literature, and indeed, this dichotomy is the conceptual foundation of Dalton’s engaged/duty-based citizenship types.

Many challenges arise in attempts to categorize different kinds of political participation (Fox 2014; Van Deth 2014), and two main challenges characterize efforts to empirically study the electoral/non-electoral dichotomy that is often implicitly referenced in the literature. The first challenge is the difficulty of definitively categorizing political acts in only one of these two groups. Although certain acts can be easily categorized (e.g., voting is a duty-based behavior while protesting is an engaged behavior), the appropriate categorization of actions as “conventional” or “unconventional” changes as political conventions shift (Brady 1999, p. 768; Hooghe 2014; Norris et al. 2005; Teorell et al. 2007, p. 343; Van Aelst and Walgrave 2001). An additional challenge of identifying types of political participators based on these two categories of political acts is the pervasiveness of a “political activities” approach, which Brady (1999, p. 794) described as “the mainstay of all participation studies.” The empirical focus of the political activities approach is the analysis of single political acts (or series of acts) based on respondents’ answers to survey items on a questionnaire (Sinclair-Chapman et al. 2009, p. 552).

Yet, the theoretical argument that motivates the transformational school of thought does not focus on separate political acts, but rather on how individual actors engage in electoral-oriented acts and simultaneously refrain from non-electoral participation, and vice versa. An empirical examination of the implications of the transformational school of thought for patterns in citizen participation would therefore require shifting the level of analysis from *political acts* as separate items on a questionnaire to *political actors* who combine these items in specific ways. To develop a research design that investigates types of political participators, we propose that Harris and Gillion’s (2010) metaphor of a “political toolbox” is a useful conceptual framework. Harris and Gillion (2010, p. 151) used the toolbox metaphor to recognize that individual actors may simultaneously engage in different categories of political action. A research design that operationalizes this insight would therefore focus on how participants combine a variety of political acts in their personal toolboxes of political action to identify what Norris (2002: p. 222, 2007: p. 640) referred to as “mixed-action” repertoires in which individuals combine electoral activities and protest politics in various ways—rather than following the common approach of analyzing separate political acts or indices of acts.

Although the theoretical discussion of ideal types of participators who combine political acts in specific ways is not new to the literature, there have been relatively few studies that have operationalized this theoretical approach (Amnå and Ekman 2014; Barnes et al. 1979; Johann 2012; Verba and Nie, 1972). An example of a prominent early empirical investigation of distinctive types of political participants is Verba and Nie’s (1972) research on participation in the United States, and the technical difficulties of this kind of analysis were reviewed by the authors.^3^ Verba and Nie noted that the factor analysis and correlational techniques they used to achieve other goals of the study were not appropriate for identifying specialist types of participators, because these techniques are “directed to the classification of *variables* and not to the classification of *people*” (Verba and Nie 1972, p. 74, emphasis in the original). To identify types of participators the authors used hierarchical cluster analysis, “a technique that typologizes people, not variables” (Verba and Nie 1972, p. 76), and thereby identified types of participators who were distinctive due to their engagement in specific combinations of political activities, and not simply because they engaged in a greater (or lesser) total number of political acts. As noted by Verba and Nie (1972, pp. 390–402), the clustering technique used in their research had important technical limitations regarding the lack of objective criteria for model choice, and subsequent prominent studies in the field have not used this approach.

Although the clustering technique used in the current analysis (detailed below) differs from that used in Verba and Nie’s (1972) study, the basic analytical goal is the same: to empirically identify distinct types of participators, including “specialists” in certain kinds of activities, based on a broad range of opportunities for civic and political action.

### Hypotheses

Two hypotheses regarding expected types of political participators emerge from the literature on the implications of changing citizenship norms for patterns of citizen participation:

*Types of Political Participators (H1)*

#### **H1a**

Engaged participator: An engaged participator type will be active beyond the electoral arena (e.g. protest), and relatively inactive in institutionalized political behavior (e.g. voting).

#### **H1b**

Duty-based participator: A duty-based participator type will be active in electoral-oriented political behavior (e.g. voting), and relatively inactive in non-institutionalized participation (e.g. protest).

Previous research suggests a second set of hypotheses regarding the citizenship norms and the socio-demographic characteristics that will be associated with these predicted participator types. Scholars who view the emergence of engaged citizenship as a sign of political renewal also recognize that this new style of political action is relatively demanding, and therefore more common among those who are socio-economically advantaged (Dalton 2000, pp. 929–930; Norris 2002; Sloam 2014). While a pure post-industrial values-change argument proposes that the rising tide of education and values change will eventually lift all boats, scholars such as Dalton and Norris have noted that, in line with extensive work on political voice and inequality (e.g.,. Marien et al. 2010; Verba et al. 1995), this new style of engagement may be accompanied by increased participatory gaps along socio-economical lines. The literature also suggests that young people are particularly likely to be engaged participants. Finally, adherence to engaged citizenship norms is expected to be correlated with non-electoral political behavior, while adherence to duty-based norms is expected to be correlated with electoral-oriented political behavior.

*Socio*-*Demographic and Normative Characteristics of Participator Types (H2)*

#### **H2a**

Socio-economic advantage: Socio-economically advantaged actors are likely to belong to participator types that emphasize non-institutionalized political behavior.

#### **H2b**

Age: Young citizens are likely to belong to participator types that emphasize non-institutionalized political behavior.

#### **H2c**

Norms and behavior: Adherence to an engaged citizenship norm will be a determinant of participator types that emphasize non-institutionalized political behavior. Conversely, adherence to a duty-based citizenship norm will be a determinant of participator types that emphasize electoral-oriented political behavior.

## Data and Methods

### Data

To test these hypotheses we analyze data from the United States “Citizenship, Involvement and Democracy” (CID) survey conducted by Georgetown University’s Center for Democracy and Civil Society. The survey was conducted between May 16 and July 19, 2005 and covered a wide variety of topics related to American political behavior and values (Howard et al. 2005). International Communications Research (ICR) conducted 1001 in-person interviews with adults aged 18 and older using a nationally representative sample design (see the “Appendix” for survey documentation). All respondents were asked whether they had participated in 15 specific political acts in the previous 12 months, including whether they voted in the 2004 national election.

**Fig. 1:**
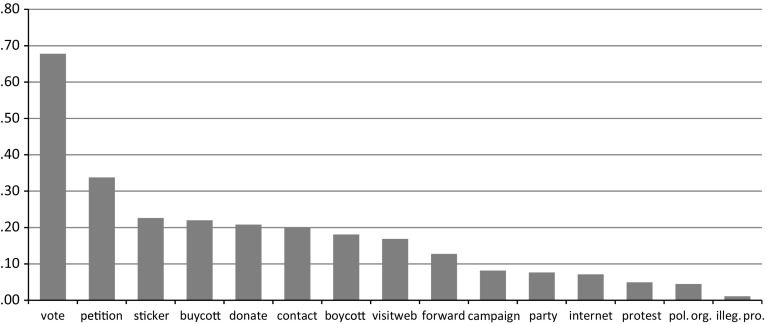
Mean prevalence of political acts in the United States in 2005 *Source*: U.S. CID Survey, n = 1001. See the “Appendix” for further survey details

The U.S. CID is a particularly useful dataset for examining the relationship between citizenship norms and political behavior because it includes a uniquely extensive battery of questions on citizenship norms along with a wide variety of political activities, including both traditional electoral activities and more recently prevalent acts beyond the electoral arena. This type of data gathering about a broad range of civic and political behavior from a single sample of respondents is fairly unusual, as many of the leading surveys on the topic focus exclusively on either electoral-oriented or non-institutionalized forms of participation (Kittilson 2007; Norris 2008), or have a relatively limited number of indicators (e.g., the International Social Survey Program’s 2004 module on citizenship analyzed by Bolzendahl and Coffé 2013). Another important advantage of the U.S. CID data for the current study’s aim of operationalizing an actor-centered measurement model is that the results concerning participator types can be readily compared to previously published findings (Dalton 2008) that use the same data to analyze single political acts. Figure 1 presents the mean prevalence of each political act included in the U.S. CID survey in 2005, ranging from the most popular act of voting (reported by 68 % of respondents) to the least common act of illegal protest (reported by 1 %).

### Methods

The participator types described in the hypotheses can be understood as discrete groups of citizens who score high on some indicators (e.g. electoral-oriented acts) while simultaneously scoring low on others (e.g. non-institutionalized acts). Latent class analysis (LCA) is particularly well suited to examine precisely this kind of typological construct through the empirical identification of discrete clusters of respondents who share a similar combination of responses across a battery of indicators (Goodman 2007; Hagenaars and Halman 1989; McCutcheon 1987).^4^ In what has been described as a “person-oriented approach” (Collins and Lanza 2010, p. 8), finite mixture models such as LCA are widely used in the social and health sciences to identify individual membership in such groups (Magidson and Vermunt 2004). Although this type of a finite mixture model measurement approach has not been widely used in the study of citizen participation, LCA has become common in social and political research that seeks to identify subgroups of the population whose members share characteristics, including studies identifying different types of revolutionary groups (Beissinger 2013), party support (Breen 2000), cultural consumption (Chan and Goldthorpe 2007; Zavisca 2005), citizenship norms (Hooghe and Oser 2015; Hooghe, Oser and Marien 2016; Oser and Hooghe 2013), tolerance and conformity (McCutcheon 1985), media use types (Lei 2011), participator types (Oser et al. 2014) and human development (Owen and Videras 2015).

From a technical perspective, LCA is similar to standard variants of cluster analysis in that it identifies distinct subgroups in the research population that share a similar response pattern on a series of items (Hagenaars and McCutcheon 2002). The probabilistic estimation method used in LCA ameliorates a main drawback of traditional cluster analysis, however, because LCA yields objective goodness of fit indicators that provide reliable criteria for determining the optimal number of latent classes (Raftery 1995; Vermunt and Magidson 2002).

## Results

### Types of Participators (H1)

Among the goodness of fit statistics used to identify optimal solutions, the Bayesian Information Criterion is the most widely used, with lower values indicating better model fit (Nylund et al. 2007; Yang 2006). The model fit statistics in Table 1, which begin with a one-cluster model and add subsequent clusters to assess model fit, show that a four-class model provides optimal fit to the data.

**Table 1:** LCA model fit statistics for participant types. *Source*: U.S. CID 2005, n = 966

	LL	BIC (LL)	L^2^	Class. Err.
1-Cluster	−5673	11,449	3961	0.00
2-Cluster	−4657	9527	1928	0.03
3-Cluster	−4505	9333	1625	0.05
4**-**Cluster	**−4445**	**9323**	**1505**	**0.08**
5-Cluster	−4399	9341	1413	0.08
6-Cluster	−4360	9373	1335	0.10

Entries are test statistics for latent class models identifying one and more clusters of respondents. Optimal model marked in bold

*BIC* Bayesian Information Criterion, *LL* log likelihood, *L* ^*2*^ likelihood ratio Chi square statistic

**Fig. 2:**
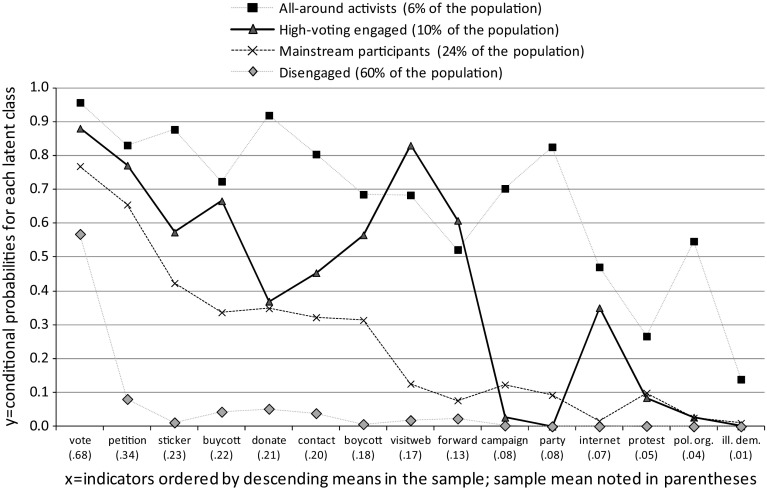
Four types of political participators identified by latent class analysis *Source*: Latent class analysis of U.S. CID 2005, n = 966

The four types of participators identified by LCA are presented in Fig. 2. Participation indicators are along the x-axis in descending order of the sample mean (noted in parentheses beneath the x-axis), and the y-axis measures the probability that members of a particular latent class engage in each political act. The markers represent the probability that members of a latent class are active in each political act, and the connecting lines aid in discerning the combination of acts that is distinctive to each latent class. If the participator types differed merely in their degree of overall activity, but not in their distinctive combination of acts, the latent classes would yield conditional probabilities parallel to the sample mean. As Fig. 2 shows, however, the identified participator types are characterized by distinctive combinations of acts.

The group referred to as “all-around activists” is distinguished by a high likelihood of engaging in all opportunities for political action, and is the smallest group (6 % of the sample population). This group has the highest probability of engaging in the most common political act (voting: .96 probability compared to the sample mean of .68) as well as the least common act (illegal protest: .14 probability compared to the sample mean of .01). These all-around activists are relatively likely to engage in activities that the three other groups essentially avoid, including political campaigning, party membership, protest, and joining a political organization. In sum, all-around activists are distinctive in their tendency to include a diverse range of political acts—including electoral as well as non-electoral—in their personal toolboxes of political participation.

Only one group rivals the all-around activists on any political act, namely the group labeled “high-voting engaged,” which includes 10 % of the population. This group has a relatively high likelihood of activity in “direct action” behaviors, such as signing a petition, political consumerism activities and online political engagement. At the same time, this group essentially does not participate in other electoral-oriented activities such as campaign and party activity, and is about on par with the population mean for protest activities. The group’s pattern of political engagement is therefore in many ways consistent with Dalton’s (2008) description of an “engaged” participant. However, given the emphasis in the transformational school’s theory on how citizens combine the act of voting with other political acts, it is noteworthy that this group has a very high probability of voting (.88 compared to the .68 sample mean).

Two remaining groups round out the empirical picture. The group labeled “mainstream participants” (24 % of the population) has a pattern of political engagement that generally reflects the relative magnitudes of the sample means of the political acts. Finally, the group labeled “disengaged,” which includes 60 % of the population, has the lowest probability of participating in all opportunities for political action. A more precise description of this group would be “low-voting disengaged” since some members of this group do vote. It is noteworthy, however, that the overall likelihood of voting in this group is lower than in any other participator type, and lower than the overall sample mean. Beyond voting, this disengaged group has very low probabilities of participating in all other political acts and its members are essentially inactive in political activities that are least common in the population as a whole.

**Fig. 3:**
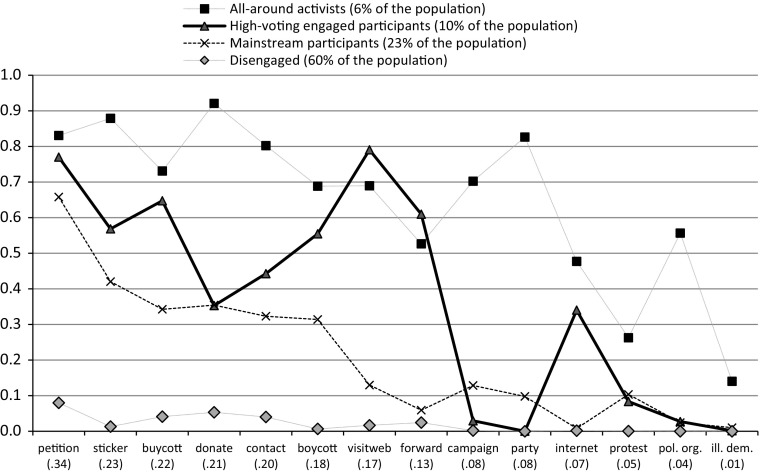
Four types of participants identified by latent class analysis parallel findings to Fig. 2, but excluding the ‘voting’ indicator from the analysis *Source*: Latent class analysis of U.S. CID 2005, n = 966

The question naturally arises as to whether the findings change in meaningful ways when the act of voting is excluded from the analysis, due to its high prevalence in comparison to other political acts. As shown in the “Appendix” (Fig. 3), when voting is excluded from the model the results are almost identical to those presented here: the same four types of participants are identified, and the disengaged group (which still encompasses 60 % of the population) remains disengaged from the broad range of political opportunities included in the analysis.

In sum, the findings partially confirm H1a, the “engaged participator” hypothesis via the identification of the “high-voting engaged” group. The presence of this group confirms expectations in the literature that a group of citizens that is rarely involved in party and campaign activity is robustly active in other political behaviors. This group deviates, however, from the ideal type of the engaged participator as described in the literature in one important way: its members are highly likely to vote. The “duty-based participator” hypothesis (H1b) is not confirmed. The findings yield no evidence of a distinct group of citizens who are highly engaged in electoral-oriented political activity, but disengaged from other political acts. Although the “all-around activist” group identified in this analysis does not receive attention in the transformational school of thought, the existence of this kind of group is suggested by a number of prior studies using a variety of methodologies that provide evidence of high correlation between electoral-oriented and non-institutionalized activity (Baek 2010; Marsh and Kaase 1979; Marsh et al. 2007; Norris et al. 2005; Saunders 2014; Stolle et al. 2005; Teorell et al. 2007; Van Aelst and Walgrave 2001). The existence of this small all-around activist group along with the large disengaged group clarifies that the transformational school of thought’s argument about renewed political action must also take into account that a small group of activists engage in all possible political acts, while a large group of citizens are largely disengaged.

### Normative and Socio-Demographic Characteristics of Participator Types (H2)

Building on the latent class analysis findings in the previous section, we now examine citizenship norms and socio-demographic characteristics as determinants of membership in the identified participator types. To facilitate comparisons to prior findings, we use the same control variables as Dalton (2008), but a different dependent variable. In contrast to Dalton’s approach of using separate political acts as dependent variables in a series of separate ordinary least squares (OLS) regression analyses, we use respondents’ membership in the participator types identified by LCA as a four-category dependent variable in a multinomial logistic regression.

To contextualize the current findings we first review the results of Dalton’s OLS regression analyses of the separate political acts. The independent variables in Dalton’s model included respondents’ factor scores on engaged and duty-based citizenship norms, as well as a number of common demographic controls, namely education, gender, age, and race (African-American and Hispanic). Dalton’s analysis of separate political acts indicated that adherence to a duty-based citizenship norm is positively related to only two political acts (voting and displaying campaign material), while adherence to an engaged citizenship norm had a positive relationship with every political act in the study except voting. Based on these findings, Dalton made a generational change argument that the decline of duty-based norms (which are more common among the older cohort) may erode turnout in elections, while the spread of engaged citizenship norms (which are more common among the younger cohort) may stimulate participation “especially in new forms of activity outside of the electoral arena” (2008, p. 88).

Table 2 presents the findings of multinomial logistic regression models that use the respondents’ participator type as a four-category dependent variable. Mirroring Dalton’s (2008) analysis, the citizenship norms are operationalized as the respondents’ scores on duty-based and engaged norms based on the factor analysis reported in Dalton’s research.^5^ Education was reported as highest grade completed, ranging from (0) eighth grade or less to (6) post-graduate training (*M* = 3.2, *SD* = 1.58). Age was reported in years *(M* = 44.9, *SD* = 16.86, range 18–90). Gender was coded ‘1’ for male (43.66 % male), and race was controlled for with a dichotomous variable for black (15.68 % of the sample) and Hispanic (9.59 % of the sample). Descriptive statistics are presented in the “Appendix” (Table 5). In Table 2, an odds ratio greater than one indicates that the control variable increases an individual’s likelihood of belonging to that type relative to their likelihood of belonging to the reference category (mainstream participants), whereas an odds ratio less than one indicates that the odds decline. An odds ratio statistically indistinguishable from 1 suggests no significant difference in comparison to the reference group.

**Table 2:** Participant type characteristics: multinomial logistic regression analysis. *Source*: U.S. CID 2005 (n = 911)

	Disengaged citizens		High-voting engaged participants		All-around activists	
	B (SE)	Odds ratio	B (SE)	Odds ratio	B (SE)	Odds ratio
Duty-based norm	−0.236** (0.100)	0.790	0.039 (0.157)	1.040	−0.342** (0.162)	0.710
Engaged norm	−0.532*** (0.091)	0.588	0.029 (0.141)	1.029	0.480*** (0.174)	1.617
Education	−0.253*** (0.056)	0.777	0.457*** (0.093)	1.579	0.290*** (0.103)	1.337
Age	−0.010* (0.005)	0.990	−0.034*** (0.009)	0.967	−0.002 (0.009)	0.998
Male	−0.317* (0.170)	0.728	0.098 (0.252)	1.103	0.290 (0.299)	1.337
Black	0.229 (0.242)	1.258	−0.358 (0.400)	0.699	−0.390 (0.467)	0.677
Hispanic	0.480 (0.321)	1.616	−0.449 (0.518)	0.638	−0.475 (0.670)	0.622
Intercept	2.198*** (0.346)		−1.128** (0.540)		−2.576*** (0.662)	
Pseudo R^2^ = 0.115						

Reference category: Mainstream participants. Entries are coefficients with standard errors in parentheses

Sign. *** *p* < .001;** *p* < .01; * *p* < .05

A more precise estimation of the effect of citizenship norms on participant types that does not rely on comparison to a reference group is found in Tables 3 and 4. These tables display the predicted probabilities of belonging to the political participant types across different levels of duty-based and engaged citizenship norms. Probabilities were calculated by varying the norms scales from 1.5 standard deviations below to 1.5 standard deviations above the average while setting race to white, and gender to male, and holding all other variables at their means. The tables also display 95 % confidence intervals estimated using simulation via the Clarify Stata module (Tomz et al. 2003).

**Table 3:** Predicted probabilities of participant types by levels of duty-based norms

	1.5 SD below mean	1.5 SD above mean
Pr(disengaged)	0.604 [.521, .683]	0.469 [.387, .552]
Pr(mainstream)	0.226 [.166, .298]	0.348 [.271, .430]
Pr(high-voting engaged)	0.078 [.043, .126]	0.131 [.080, .197]
Pr(all-around activist)	0.091 [.051, .150]	0.052 [.027, .089]

The first column of results shows the probabilities for respondents with duty-based norms scores that are 1.5 standard deviations below the mean; the subsequent column displays the probabilities for respondents with duty-based scores that are 1.5 standard deviations above the mean. 95 % confidence intervals in brackets estimated using simulation via the Clarify Stata module (Tomz et al. 2003). Probabilities for white males with other variables set at their means

**Table 4:** Predicted probabilities of participant types by levels of engaged citizenship norms

	1.5 SD below mean	1.5 SD above mean
Pr(disengaged)	0.745 [.677, .804]	0.313 [.245, .390]
Pr(mainstream)	0.174 [.126, .233]	0.367 [.289, .449]
Pr(high-voting engaged)	0.06 [.033, .098]	0.136 [.085, .203]
Pr(all-around activist)	0.021 [.009, .042]	0.184 [.112, .274]

The first column of numbers shows the probabilities for engaged citizenship norms scores that are 1.5 standard deviations below the mean; the subsequent column displays the probabilities for citizens with engaged citizenship scores that are 1.5 standard deviations above the mean. 95 % confidence intervals in brackets estimated using simulation via the Clarify Stata module (Tomz et al. 2003). Probabilities for white males with other variables set at their means

The findings for the socio-demographic control variables are consistent with previous research. As predicted (H2a), those with socio-economic advantages in terms of education are more politically active. The results also confirm the expectation (H2b) that younger individuals are more likely to belong to the participant type that is most similar to the expected activity of engaged participants (i.e., the high-voting engaged group).

The findings regarding the relationship between citizenship norms and participant types (H2c), however, differ in two important ways from the expectations in the research literature. First, citizens who adhere to the normative emphases expected of the ideal type of “engaged participators” (i.e., high levels of engaged norms and low levels of duty-based norms) are in fact most likely to be all-around activists who participate in all political opportunities, including electoral-oriented political acts. Second, the possibility is not discussed in the literature that a large group of citizens would have low levels of both duty-based and engaged norms, even though the current analysis shows that this is an accurate description of the normative determinants of the large “disengaged” group.

In sum, the actor-centered analysis of socio-demographic determinants of the participator types identified by LCA confirm expectations in the literature regarding age and education, but add important caveats to expectations regarding citizenship norms adhered to by different types of political participators. These findings do not invalidate Dalton’s (2008) OLS findings of the determinants of separate political acts. Rather, the findings in the current study clarify that when research designs analyze separate political acts, the findings are reflective of the political behavior of individual survey respondents who may be simultaneously active in a variety of electoral and non-electoral political acts.

## Discussion

In this study we clarify that in research on new kinds of engaged citizenship norms and citizen participation, an important theoretical construct of interest is how actors combine separate political acts—and not only the separate political acts themselves. We then use latent class analysis to empirically investigate the existence of different types of political participators, each of which combines political behaviors in a distinct way. In this section we review some of the methodological and substantive implications of the findings in this study for research on changing patterns of citizen participation in advanced democracies.

Three main findings from the current study enrich prior research on citizenship norms and citizen participation. First, the findings regarding participator types partially confirm expectations in the literature, while raising questions for future research. A duty-based type of participator that specialized in electoral-oriented political acts was not identified. While an engaged type of participant that emphasized non-institutionalized participation was identified, this group unexpectedly has a very high probability of voting. Because the “high-voting engaged” group had a higher probability of voting than the sample mean, other explanations are therefore needed for the decline or stagnation in turnout rates noted by Dalton and others. The identification of two additional participator types in the current study that do not receive attention in the literature on changing citizenship norms—namely a small group of all-around activists and a large group of disengaged citizens—highlights a new perspective that emerges when we shift the measurement approach from *political acts* as separate items on a questionnaire to *political actors* who combine these items in specific ways.

Second, the findings regarding the normative determinants of these participator types do not support the argument that a replacement over time of duty-based norms by engaged norms will lead to an expansion of political participation for those who uniquely specialize in non-electoral politics. The participator type that adheres to norms most similar to those that Dalton predicted for “engaged citizenship”—i.e., the group that has relatively high levels of engaged citizenship norms but low levels of duty-based norms—is paradoxically the “all-around activist” participator type whose members are most likely to be active in all political activities, including electoral-oriented acts. This means that instead of a substitution process proposed in the literature, whereby one type of participatory specialist (e.g. engaged) will replace an opposing type (e.g. duty-based) over time, the findings indicate an expansion of action repertoires, but only for a small group of all-around activists. These findings therefore suggest that one potential implication of a generational replacement of the duty-based norm by an engaged citizenship norm would be an increase in the proportion of “all-around activists” who take advantage of all political opportunities available, including the various forms of internet activism that have increasingly gained attention of participation scholars (Bode 2012; Gainous and Wagner 2014; Gibson and Cantijoch 2013; Oser et al. 2013; Wells 2015). While some may view the possibility that the rise in engaged norms will increase the size of the small “all-around activist” group as a positive development from the perspective of democratic engagement, this finding strengthens observations in the literature that norm change may increase various aspects of participatory inequality (Dalton 2000, pp. 929–930; Norris 2002; Sloam 2014).

Third, the findings in the current study regarding the disengaged group caution against claims that norm change and/or the proliferation of new kinds of political acts will, on their own, lead to a generalized civic and political renewal. The optimistic tone of recent research on the expansion of political participation suggests an increased citizen influence (Dalton 2008, p. 94), and terms like Norris’s (2002) “democratic phoenix” can leave the impression that the proliferation of new kinds of political acts has invigorated civic democracy writ large. The current study’s focus on how participators combine acts in their political toolbox clarifies, however, that voting levels in the U.S. in the 2004 national election were not depressed by the presence of an engaged group of young participants who uniquely emphasized political action beyond the voting booth. The evidence instead indicates that among those who vote are also individuals who adhere to engaged citizenship norms and have expanded their personal repertoire of political participation to include new political opportunities as they arise.

In order to understand the implications of changing citizenship norms and participation for democratic mass publics as a whole, it would seem that more attention must be paid to the low-voting disengaged group—a full 60 % of the U.S. population in 2005—whose members do not take advantage of the many increasingly prevalent acts of political engagement. For this purpose, studies such as Han’s (2016) investigation of organizational cultivation of political activism in the U.S. are needed to investigate the ways in which new activists can be recruited and developed in different contexts. In addition, as noted in recent research on “standby citizens,” more nuanced research is needed on the large group of non-participators in contemporary democracies in order to bring further evidence to bear regarding the normative debate as to whether political passivity can be considered an “asset or threat” to democracy (Amnå and Ekman 2014, 261). The findings in the present research therefore highlight the importance of an actor-centered measurement approach in order to systematically examine the precise ways, and for whom, democratic participation is expanding due to norm change.

Taken together, these substantive contributions highlight the importance of better understanding changing patterns in norms and participation to clarify the implications for the health and quality of contemporary democracies (Morlino 2011). It is noteworthy, however, that the substantive contributions of the current study’s findings are facilitated by the research design’s comparison to extant research. Despite this important advantage of the research design, there are two caveats to the generalizability of the reported findings. First, as a single-country study, this article clarifies the need to continue to develop more comprehensive and expansive cross-national data gathering efforts in order to build upon comparative research on these topics. Particularly since the U.S. context is considered exceptional due to distinctive political culture characteristics and unique political institutions, future research will need to test the generalizability of the findings reported in the present study. Second, as a cross-sectional analysis, the present study cannot determine whether the relationship between norms and participation patterns is causal, and therefore the use of latent transitional analysis (LTA) with longitudinal and panel data will be required to assess causality (Lanza et al. 2013). Further research is required to determine how the political participator types identified in the current study may vary over time. While the limited availability of long-term longitudinal data on attitudes and political participation is well known (Blais and Rubenson 2013), particularly outside of electoral participation, future research on this topic will benefit from the recent development of several high-quality longitudinal data sets in a number of countries such as Belgium (Hooghe et al. 2015) and Sweden (Amnå et al. 2009).

From a measurement perspective, however, the findings shed new light on the advantage of using an actor-centered measurement approach when the theoretical interest of the researcher is to understand how political actors combine a variety of acts in their “political tool box” of participation. The findings clarify that the “voting” population, for example, encompasses a number of different types of political participators when their broader toolbox of political engagement is taken into account. With research proving that online or “e-participation” indeed constitutes a distinct dimension of political participation (Gibson and Cantijoch 2013; Oser et al. 2013), a recent wave of research has emerged to investigate what has been referred to as a “new digital repertoire of contention” (Earl and Kimport 2011, p. 177). Given the keen interest in the expansion of new types of online and social media activism indicators, the actor-centered measurement approach used in this article enables the researcher to investigate how new types of participation are (or are not) adapted by different types of political actors.

## Appendix

### U.S. CID 2005 Survey

Prefatory question: “During the last 12 months, have you done any of the following?”

Listed in order of descending means, as presented in Fig. 1.

*Vote*: “Did you get a chance to vote in the 2004 presidential election when George W. Bush and John Kerry were running for president?”
*Petition*: “Signed a petition”
*Sticker*: “Worn or displayed a campaign badge/sticker”
*Buycott*: “Deliberately bought certain products for political, ethical, or environmental reasons”
*Donate*: “Donated money to a political organization or group”
*Contact*: “Contacted a politician, government or local government official”
*Boycott*: “Boycotted certain products”
*Visitweb*: “Visited websites of political organizations or candidates”
*Forward*: “Forwarded electronic messages with political content”
*Campaign*: “Worked for the campaign of a candidate for office”
*Party*: “Worked in a political party or action group”
*Internet*: “Participated in political activities over the internet”
*Protest*: “Taken part in a lawful public demonstration”
*Pol. org*: “Worked in another [not electoral campaign-related] political organization or association”
*Illeg.**pro.*: “Participated in illegal protest activities”

See Tables 5, 6.

**Table 5:** Descriptive statistics. *Source*: U.S. Citizenship, Involvement and Democracy survey

	By participator type				
	Disengaged citizens (n = 573)	Mainstream participants (n = 232)	High-voting engaged (n = 99)	All-around activists (n = 62)	Total
Duty norm (mean)	−0.05	0.16	0.07	−0.14	0.01
Engaged norm (mean)	−0.28	0.22	0.27	0.58	−0.05
Education (mean)	2.82	3.44	4.37	4.16	3.22
Age (mean)	44.0	47.1	39.9	45.8	44.9
% Male	40.7	46.1	45.5	51.6	43.7
% Black	16.7	14.7	10.1	12.9	15.7
% Hispanic	11.7	8.2	6.1	4.8	9.6

See footnote 5 for further information on the duty norm and engaged norm data

**Table 6:** LCA model fit statistics for participant types. *Source*: U.S. CID 2005, n = 966

	LL	BIC(LL)	L^2^	Class.Err.
1-Cluster	**−**5060	10,217	3643	0.00
2-Cluster	**−**4079	8357	1679	0.03
3-Cluster	**−**3932	8167	1387	0.05
4-Cluster	**−3872**	**8150**	**1267**	**0.08**
5-Cluster	**−**3832	8173	1186	0.09
6-Cluster	**−**3795	8203	1113	0.10

Parallel findings to Table 1, but excluding the ‘voting’ indicator from the analysis. Entries are test statistics for latent class models identifying one and more clusters of respondents

Preferred model marked in bold

*BIC* Bayesian Information Criterion, *LL* log likelihood, *L* ^*2*^ likelihood ratio Chi square statistic

## Footnotes

1 Additional terms used to address overlapping aspects of this explicit or implicit dichotomous distinction in the literature include a loose division between “old” and “new” participation (e.g. Norris 2002), “elite-directed” versus “elite-challenging” acts (Inglehart and Catterberg 2002), and “electoral engagement” versus “political voice” (Zukin et al. 2006). For the sake of simplicity we reference terms most commonly used to describe this dichotomous distinction.

2 Verba et al. (1978) four main modes of participation included voting, campaign activity, communal activity and particularized contacts. Teorell et al. (2007) five modes of participation included voting, party activity, consumer participation, contacting and protest activity. The four dimensions in Talò and Mannarini’s (2015) study included formal political participation, activism, civic participation and disengagement.

3 One of Verba and Nie’s theoretical goals was to determine whether a broad variety of political acts were “concentrated in the hands of the few or dispersed through a wide segment of the American citizenry” (1972, p. 33). In addition to identifying “activists” who were engaged in a wide range of activities and “inactives” who were generally disengaged, Verba and Nie’s analysis found that political acts were also dispersed in the hands of specialist types of activists, such as “campaigners” who participated in some types of activities but not others (Verba and Nie 1972, pp. 73–81).

4 The theoretical foundations of LCA as a statistical technique were developed already in the 1940 s by Lazarsfeld (1950), and an early use of latent class modeling was to identify latent attitudes (Lazarsfeld et al. 1968). The recent availability of efficient software to implement LCA has contributed to the increased prevalence of its use (on the historical development of the technique see e.g., Goodman 2002, 2007).

5 Dalton’s reported factor analysis is the source of the “duty-based” and “engaged” normative distinction and the resulting factor scores are the variables used in the analysis reported in the current study (available in the U.S. CID dataset; variable names: “engagcit” for the engaged citizen factor score; “citduty” for the citizen duty factor score). These scores are based on Dalton’s 2008 (pp. 80–81) factor analysis of dimensions of democratic citizenship that analyzed a range of normative indicators that represent theoretical topics of autonomy, engagement, social order and solidarity.

## References

[CR1] Amnå E (2012). How is civic engagement developed over time? Emerging answers from a multidisciplinary field. Journal of Adolescence.

[CR2] Amnå E, Ekman J (2014). Standby citizens: Diverse faces of political passivity. European Political Science Review.

[CR3] Amnå E, Ekman J, Kerr M, Stattin H (2009). Political socialization and human agency: The development of civic engagement from adolescence to adulthood. Statsvetenskaplig tidskrift.

[CR4] Arendt H (1958). The human condition.

[CR5] Baek YM (2010). To buy or not to buy: Who are political consumers? What do they think and how do they participate?. Political Studies.

[CR6] Barber BR (2003). Strong democracy: Participatory politics for a new age.

[CR7] Barnes SH, Kaase M (1979). Political action: Mass participation in five Western democracies.

[CR8] Beissinger MR (2013). The semblance of democratic revolution: Coalitions in Ukraine’s Orange Revolution. American Political Science Review.

[CR9] Bennett WL (2008). Changing citizenship in the digital age. Civic life online: Learning how digital media can engage youth.

[CR10] Bennett WL (2012). The personalization of politics: Political identity, social media, and changing patterns of participation. The ANNALS of the American Academy of Political and Social Science.

[CR11] Bennett WL, Wells C, Freelon D (2011). Communicating civic engagement: Contrasting models of citizenship in the youth web sphere. Journal of Communication.

[CR12] Blais Andre, LeDuc L, Niemi RG, Norris P (2010). Political Participation. Comparing democracies 3: Elections and voting in the 21st Century.

[CR13] Blais A, Gidengil E, Nevitte N (2004). Where does turnout decline come from?. European Journal of Political Research.

[CR14] Blais A, Rubenson D (2013). The source of turnout decline: New values or new contexts?. Comparative Political Studies.

[CR15] Bode L (2012). Facebooking it to the polls: A study in online social networking and political behavior. Journal of Information Technology & Politics.

[CR16] Bolzendahl C, Coffé H (2013). Are ‘good’ citizens ‘good’ participants? Testing citizenship norms and political participation across 25 nations. Political Studies.

[CR17] Bowler S, Donovan T (2013). Civic duty and turnout in the UK referendum on AV: What shapes the duty to vote?. Electoral Studies.

[CR18] Brady H, Robinson JP, Shaver PR, Wrightsman LS (1999). Political participation. Measures of political attitudes.

[CR19] Breen R (2000). Why is support for extreme parties underestimated by surveys? A latent class analysis. British Journal of Political Science.

[CR20] Chan Tak W, Goldthorpe John H (2007). Social status and newspaper readership. American Journal of Sociology.

[CR21] Christensen HS (2014). All the same? Examining the link between three kinds of political dissatisfaction and protest. Comparative European Politics.

[CR22] Coffé H, van der Lippe T (2010). Citizenship norms in Eastern Europe. Social Indicators Research.

[CR23] Collins, L. M., & Lanza, S. T. (2010). *Latent class and latent transition analysis*: *With applications in the social, behavioral, and health sciences.* New York: Wiley.

[CR24] Copeland L (2014). Conceptualizing political consumerism: How citizenship norms differentiate boycotting from buycotting. Political Studies.

[CR25] Copeland L (2014). Value change and political action: Postmaterialism, political consumerism, and political participation. American Politics Research.

[CR26] Dahl RA (2006). On political equality.

[CR27] Dalton RJ, Pharr SJ, Putnam RD (2000). Value change and democracy. Disaffected democracies: What’s troubling the trilateral countries?.

[CR28] Dalton RJ (2008). Citizenship norms and the expansion of political participation. Political Studies.

[CR29] Dalton RJ (2015). The good citizen: How a younger generation is reshaping American politics.

[CR30] Dalton R, Shin DC, Dalton R, Welzel C (2014). Reassessing the *Civic Culture* model. The civic culture transformed: From allegiant to assertive citizens.

[CR31] Dalton RJ, Wattenberg MP (2000). Parties without partisans: Political change in advanced industrial democracies.

[CR32] Dalton R, Welzel C (2014). The civic culture transformed: From allegiant to assertive citizens.

[CR33] Earl J, Kimport K (2011). Digitally enabled social change: Activism in the internet age.

[CR34] Ekman J, Amnå E (2012). Political participation and civic engagement: Towards a new typology. Human Affairs.

[CR35] Fieldhouse E, Tranmer M, Russell A (2007). Something about young people or something about elections? Electoral participation of young people in Europe: Evidence from a multilevel analysis of the European Social Survey. European Journal of Political Research.

[CR36] Fox S (2014). Is it time to update the definition of political participation?. Parliamentary Affairs.

[CR37] Franklin MN (2004). Voter turnout and the dynamics of electoral competition in established democracies since 1945.

[CR38] Gainous J, Wagner KM (2014). Tweeting to power: The social media revolution in American politics.

[CR39] Geissel B (2008). Reflections and findings on the critical citizen: Civic education—What for?. European Journal of Political Research.

[CR40] Gibson R, Cantijoch M (2013). Conceptualizing and measuring participation in the age of the internet: Is online political engagement really different to offline?. The Journal of Politics.

[CR41] Goodman LA, Hagenaars JA, McCutcheon AL (2002). Latent class analysis: The empirical study of latent types, latent variables, and latent structures, and some notes on the history of this subject. Applied latent class analysis.

[CR42] Goodman LA (2007). Statistical magic and/or statistical serendipity: An age of progress in the analysis of categorical data. Annual Review of Sociology.

[CR43] Hagenaars JA, Halman LC (1989). Searching for ideal types: The potentialities of latent class analysis. European Sociological Review.

[CR44] Hagenaars JA, McCutcheon AL (2002). Applied latent class analysis.

[CR45] Han, H. (2016). The organizational roots of political activism: Field experiments on creating a relational context. *American Political Science Review, 110*(2). https://d3n8a8pro7vhmx.cloudfront.net/hahrie/pages/22/attachments/original/1446655776/han2016-final.pdf?1446655776.

[CR46] Harris F, Gillion DQ, Leighley JE (2010). Expanding the possibilities: Reconceptualizing political participation as a tool box. Oxford Handbook of American Elections and Behavior.

[CR47] Hooghe M (2014). Defining political participation: How to pinpoint an elusive target?. Acta Politica.

[CR48] Hooghe M, Dassonneville R, Marien S (2015). The impact of education on the development of political trust: Results from a five-year panel study among late adolescents and young adults in Belgium. Political Studies.

[CR49] Hooghe M, Oser J (2015). The rise of engaged citizenship: The evolution of citizenship norms among adolescents in 21 countries between 1999 and 2009. International Journal of Comparative Sociology.

[CR50] Hooghe M, Oser J, Marien S (2016). A comparative analysis of ‘good citizenship’: A latent class analysis of adolescents’ citizenship norms in 38 countries. International Political Science Review.

[CR51] Howard, M. M., Gibson, J. L., & Stolle, D. (2005). *The U.S. citizenship, involvement, democracy survey*. Washington, D.C.: Center for Democracy and Civil Society (CDACS), Georgetown University.

[CR52] Inglehart R (1997). Modernization and postmodernization: Culture, economic and political change in 43 societies.

[CR53] Inglehart R, Catterberg G (2002). Trends in political action: The developmental trends and the post-honeymoon decline. International Journal of Comparative Sociology.

[CR54] Jennings, M. K. (2015). The dynamics of citizenship norms. In T. Poguntke, S. Rossteutscher, R. Schmitt-Beck, & S. Zmerli (Eds.), *Citizenship and democracy in an era of crisis*: *Essays in honour of Jan W. van Deth* (pp. 93–112). London: Routledge.

[CR55] Johann D (2012). Specific political knowledge and citizens’ participation: Evidence from Germany. Acta Politica.

[CR56] Kittilson MC, Klingemann H-D, Dalton RJ (2007). Research resources in comparative political behavior. Oxford handbook on political behavior.

[CR57] Lanza ST, Bray BC, Collins LM, Schinka JA, Velicer WF, Weiner IB (2013). An introduction to latent class and latent transition analysis. Handbook of psychology: Research methods in psychology.

[CR58] Lazarsfeld, P. F. (1950). The logical and mathematical foundation of latent structure analysis. In S. A. Stouffer, L. Guttman, E. A. Suchman, P. F. Lazarsfeld, & J. A. Clausen (Eds.), *Measurement and prediction* (pp. 362–412, Studies in Social Psychology in World War II, Vol. IV). Princeton: Princeton University Press.

[CR59] Lazarsfeld PF, Berelson B, Gaudet H (1968). The people’s choice.

[CR60] Lei Y-W (2011). The political consequences of the rise of the Internet: Political beliefs and practices of Chinese netizens. Political Communication.

[CR61] Lewis-Beck Michael S, Jacoby William G, Norpoth Helmut, Weisburg Herbert F (2008). The American Voter revisited.

[CR62] Lijphart A (1997). Unequal participation: Democracy’s unresolved dilemma. American Political Science Review.

[CR63] Magidson J, Vermunt JK, Kaplan D (2004). Latent class models. The Sage handbook of quantitative methodology for the social sciences.

[CR64] Marien S, Hooghe M, Quintelier E (2010). Inequalities in non-institutionalised forms of political participation: A multi-level analysis of 25 countries. Political Studies.

[CR65] Marsh D, Akram S (2015). Political participation and citizen engagement: Beyond the mainstream. Policy Studies.

[CR66] Marsh D, Jones S, O’Toole T (2007). Young people and politics in the UK: Apathy or alienation?.

[CR67] Marsh A, Kaase M, Barnes SH, Kaase M (1979). Measuring political action. Political action: Mass participation in five Western democracies.

[CR68] Martin A (2012). Political participation among the young in Australia: Testing Dalton’s good citizen thesis. Australian Journal of Political Science.

[CR69] McCutcheon AL (1985). A latent class analysis of tolerance for nonconformity in the American public. Public Opinion Quarterly.

[CR70] McCutcheon AL (1987). Latent class analysis (Sage University Paper series on Quantitative Applications in the Social Sciences).

[CR71] McDonald MP, Leighley J (2010). American voter turnout in historical perspective. Oxford Handbook of American Elections and Political Behavior.

[CR72] McDonald, M. P. (2015). Presidential turnout rates, 1787–2012. United States Election Project. http://www.electproject.org/national-1789-present. Accessed January 14, 2016.

[CR73] Morlino L (2011). Changes for democracy: Actors, structures, processes.

[CR74] Norris P (1999). Critical citizens: Global support for democratic governance.

[CR75] Norris P (2002). Democratic phoenix: Reinventing political activism.

[CR76] Norris P, Boix C, Stokes S (2007). Political activism: New challenges, new opportunities. The Oxford handbook of comparative politics.

[CR77] Norris, P. (2008). The globalization of comparative public opinion research. In N. Robinson, & T. Landman (Eds.), *Handbook of comparative politics.* London: Sage.

[CR78] Norris P (2011). Democratic deficit: Critical citizens revisited.

[CR79] Norris P, Walgrave S, Van Aelst P (2005). Who demonstrates? Antistate rebels, conventional participants, or everyone?. Comparative Politics.

[CR80] Nylund KL, Asparouhov T, Muthén BO (2007). Deciding on the number of classes in latent class analysis and growth mixture modeling: A Monte Carlo simulation study. Structural Equation Modeling.

[CR81] Oser J, Hooghe M (2013). The evolution of citizenship norms among Scandinavian adolescents, 1999–2009. Scandinavian Political Studies.

[CR82] Oser J, Hooghe M, Marien S (2013). Is online participation distinct from offline participation? A latent class analysis of participation types and their stratification. Political Research Quarterly.

[CR83] Oser J, Leighley JE, Winneg KM (2014). Participation, online and otherwise: What’s the difference for policy preferences?. Social Science Quarterly.

[CR84] Owen AL, Videras J (2015). Classifying human development with latent class analysis. Social Indicators Research.

[CR85] Pateman C (1970). Participation and democratic theory.

[CR86] Pateman C (2012). Participatory democracy revisited. Perspectives on Politics.

[CR87] Phelps E (2012). Understanding electoral turnout among British young people: A review of the literature. Parliamentary Affairs.

[CR88] Quaranta M (2016). An apathetic generation? Cohorts’ patterns of political participation in Italy. Social Indicators Research.

[CR89] Raftery AE (1995). Bayesian model selection in social research. Sociological Methodology.

[CR90] Raney T, Berdahl L (2009). Birds of a feather? Citizenship norms, group identity, and political participation in Western Canada. Canadian Journal of Political Science.

[CR91] Saunders C (2014). Anti-politics in Action? Measurement dilemmas in the study of unconventional political participation. Political Research Quarterly.

[CR92] Schlozman KL, Verba S, Brady HE (2012). The unheavenly chorus: Unequal political voice and the broken promise of American democracy.

[CR93] Schudson M (1998). The good citizen: A history of American civic life.

[CR94] Schudson M (2000). Good citizens and bad history: Today’s political ideals in historical perspective. The Communication Review.

[CR95] Shehata A, Ekström M, Olsson T (2015). Developing self-actualizing and dutiful citizens: Testing the AC-DC model using panel data among adolescents. Communication Research.

[CR96] Sinclair-Chapman V, Walker RW, Gillion DQ (2009). Unpacking civic participation: Analyzing trends in black [and white] participation over time. Electoral Studies.

[CR97] Sloam J (2012). ‘Rejuvenating democracy?’ Young people and the ‘Big Society’ project. Parliamentary Affairs.

[CR98] Sloam J (2013). “Voice and equality”: Young people’s politics in the European Union. West European Politics.

[CR99] Sloam J (2014). New voice, less equal: The civic and political engagement of young people in the United States and Europe. Comparative Political Studies.

[CR100] Stolle D, Hooghe M, Micheletti M (2005). Politics in the supermarket: Political consumerism as a form of political participation. International Political Science Review.

[CR101] Talò C, Mannarini T (2015). Measuring participation: Development and validation the participatory behaviors scale. Social Indicators Research.

[CR102] Teorell J, Torcal M, Montero JR, Van Deth JW, Montero JR, Westholm A (2007). Political participation: Mapping the terrain. Citizenship and involvement in European democracies: A comparative analysis.

[CR103] Theocharis Y (2011). The influence of postmaterialist orientations on young British people’s offline and online political participation. Representation.

[CR104] Theocharis Y (2011). Young people, political participation and online postmaterialism in Greece. New Media & Society.

[CR105] Theocharis Y (2015). The conceptualization of digitally networked participation. Social Media + Society.

[CR106] Tomz M, Wittenberg J, King G (2003). Clarify: Software for interpreting and presenting statistical results. Journal of Statistical Software.

[CR107] Van Aelst P, Walgrave S (2001). Who is that (wo)man in the street? From the normalization of protest to the normalization of the protester. European Journal of Social Research.

[CR108] Van Biezen I, Mair P, Poguntke T (2012). Going, going,… gone? The decline of party membership in contemporary Europe. European Journal of Political Research.

[CR109] Van Biezen I, Poguntke T (2014). The decline of membership-based politics. Party Politics.

[CR110] Van Deth JW, Dalton RJ, Klingemann H-D (2007). Norms of citizenship. The Oxford handbook of political behavior.

[CR111] Van Deth JW, Micheletti M, McFarland A (2011). Is creative participation creative democracy?. Creative participation: Responsibility-taking in the political world.

[CR112] Van Deth, J. W. (2012). New modes of participation and norms of citizenship. In J. van Deth, & W. Maloney (Eds.), *New participatory dimensions in civil society*: *Professionalization and individualized collective action* (pp. 115–138). London: Routledge.

[CR113] Van Deth JW (2014). A conceptual map of political participation. Acta Politica.

[CR114] Van Deth JW, Montero JR, Westholm A (2007). Citizenship and involvement in European democracies: A comparative analysis.

[CR115] Verba S, Nie NH (1972). Participation in America: Political democracy and social equality.

[CR116] Verba S, Nie NH, Kim J (1971). The modes of democratic participation: A cross-national analysis.

[CR117] Verba S, Nie NH, Kim J-O (1978). Participation and political equality: A seven-nation comparison.

[CR118] Verba S, Schlozman KL, Brady HE (1995). Voice and equality: Civic voluntarism in American politics.

[CR119] Vermunt JK, Magidson J, Hagenaars JA, McCutcheon AL (2002). Latent class cluster analysis. Applied latent class analysis.

[CR120] Vráblíková K (2014). How context matters? Mobilization, political opportunity structures, and nonelectoral political participation in old and new democracies. Comparative Political Studies.

[CR121] Vromen A, Xenos MA, Loader B (2015). Young people, social media and connective action: From organisational maintenance to everyday political talk. Journal of Youth Studies.

[CR122] Wattenberg MP (2012). Is voting for young people?.

[CR123] Weber, M. (1949 [1904]). Objectivity in social science and social policy (E. A. Shils, & F. H. A., Trans.). In E. A. Shils, & F. H. A. (Eds.), *The Methodology of the Social Sciences* (pp. 49–112). New York: Free Press.

[CR124] Wells C (2014). Two eras of civic information and the evolving relationship between civil society organizations and young citizens. New Media & Society.

[CR125] Wells C (2015). The civic organization and the digital citizen: Communicating engagement in a networked age.

[CR126] Welzel C (2007). Are levels of democracy affected by mass attitudes? Testing attainment and sustainment effects on democracy. International Political Science Review.

[CR127] Welzel C (2013). Freedom rising: Human empowerment and the quest for emancipation.

[CR128] Welzel C, Deutsch F (2012). Emancipative values and non-violent protest: The importance of “ecological” effects. British Journal of Political Science.

[CR129] Whiteley Paul F (2011). Is the party over? The decline of party activism and membership across the democratic world. Party Politics.

[CR130] Yang C-C (2006). Evaluating latent class analysis models in qualitative phenotype identification. Computational Statistics & Data Analysis.

[CR131] Zavisca J (2005). The status of cultural omnivorism: A case study of reading in Russia. Social Forces.

[CR132] Zukin C, Keeter S, Andolina M, Jenkins K, Delli Carpini MX (2006). A new engagement? Political participation, civic life, and the changing American citizen.

